# Two-Stage Tissue-Expander Breast Reconstruction: A Focus on the Surgical Technique

**DOI:** 10.1155/2017/1791546

**Published:** 2017-12-10

**Authors:** Elisa Bellini, Marianna Pesce, PierLuigi Santi, Edoardo Raposio

**Affiliations:** ^1^Department of Medicine and Surgery, Plastic Surgery Section, University of Parma, Parma, Italy; ^2^Cutaneous, Mini-Invasive, Regenerative and Plastic Surgery Unit, Parma University Hospital, Parma, Italy; ^3^Plastic Surgery Chair, Department of Surgical Sciences and Related Methodologies (DICMI), University of Genova, Genova, Italy

## Abstract

**Objective:**

Breast cancer, the most common malignancy in women, comprises 18% of all female cancers. Mastectomy is an essential intervention to save lives, but it can destroy one's body image, causing both physical and psychological trauma. Reconstruction is an important step in restoring patient quality of life after the mutilating treatment.

**Material and Methods:**

Tissue expanders and implants are now commonly used in breast reconstruction. Autologous reconstruction allows a better aesthetic result; however, many patients prefer implant reconstruction due to the shorter operation time and lack of donor site morbidity. Moreover, this reconstruction strategy is safe and can be performed in patients with multiple health problems. Tissue-expander reconstruction is conventionally performed as a two-stage procedure starting immediately after mammary gland removal.

**Results:**

Mastectomy is a destructive but essential intervention for women with breast cancer. Tissue expansion breast reconstruction is a safe, reliable, and efficacious procedure with considerable psychological benefits since it provides a healthy body image.

**Conclusion:**

This article focuses on this surgical technique and how to achieve the best reconstruction possible.

## 1. Objective

With 1 million new cases in the world each year, breast cancer is the most common malignancy in women and comprises 18% of all female cancers. Breast cancer is responsible for 20% of the deaths in the female population and is the main cause of cancer in women between the ages of 40 and 55. The main epidemiological risk factors include age, age at menarche and menopause (early menarche and late menopause increase risk), age at first pregnancy (women who have their first child after age of 30 have approximately twice the risk of women who have their first child before age of 20), positive family anamnesis, previous benign breast disease, history of radiation, lifestyle factors (diet, weight, alcohol intake, and smoking), and endogenous factors (estrogen levels, genetic predisposition) [1].

In our society, breasts are integral parts of female beauty and sexuality. A woman's body image and self-esteem can be negatively affected by any alteration in her natural form. Surgery may be necessary to save a woman's life, but for many women these events can be traumatic, both physically and psychologically [2].

Unfortunately, mastectomy is a mutilating operation that, without reconstruction, causes deformity in women [3]. The general goals of breast reconstruction are to restore the missing form of the female breast as well as the location and size of the breast, so that women no longer need to wear an external prosthesis [4]. When they look in the mirror, they feel feminine and attractive and usually no longer need to go to a support group for reassurance. The ultimate goal of breast reconstruction is for a woman to return to her normal life, including both family activities and work [5].

The quality of breast reconstruction will depend not only on the surgeon's skills but also on the amount of missing tissue, patient health conditions, size of the opposite breast, and breast reconstruction technique. Obesity will make reconstruction difficult and is an independent risk factor for perioperative complications, as is smoking, hypertension, chemotherapy, radiotherapy, and age over 65 years [6, 7].

In patients with multiple health problems, reconstruction using implants may be preferred due to shorter operation duration. However, the fact that it may be a multistage operation with required revisions resulting in the patient receiving anesthesia multiple times should not be ignored [8].

Prosthetic implants began to be used for breast reconstruction in the 1960s. The first silicone prosthesis was developed in 1961 and was used in augmentation mammoplasty in 1962 [9]. Since that time, the prosthesis began to be used for reconstructions in mastectomized women [10]. Breast reconstructions using prosthetic implants were applied in a single stage at first. The development of tissue expanders by Radovan created new possibilities in immediate and delayed reconstructions, however, and the popularity of single-stage reconstruction with implants was overridden by two-stage reconstructions in the 1980s [11, 12].

Initially, the silicone implant was incorporated into breast reconstruction only to reestablish the conic shape when radical mastectomy and modified radical mastectomy were routinely performed. In these cases, the implant was always used in conjunction with flap coverage [13]. The evolution toward less destructive mastectomies in oncologic treatment allowed preservation of enough soft tissue for implant-based breast reconstruction. Changes in oncologic practice, such as an increased number of bilateral mastectomies, have contributed to the expansion of implant use [14].

With the development of the concept of tissue expansion, a deflated implant can be inserted into the new pocket created after mastectomy. The expander can subsequently undergo inflation both to stretch the dimensions of the retained skin envelope and to avoid wound contraction during the process of wound maturation after the mastectomy [15]. With improvement in the design of tissue expanders, the port is incorporated into the surface of the implant, eliminating dissection distant to the mastectomy site for valve placement and reducing remote port complications [16, 17]. Textured expanders replaced smooth-surfaced expanders to minimize capsular contracture and migration. With the use of a textured expander, the expander will not migrate away from the area of greatest skin tightness (usually the inferior half of the preserved breast skin envelope) and will maintain a well-defined inframammary line, despite mastectomy [18]. These innovations have made the expander implant for breast reconstruction a reliable technique to restore form at the mastectomy site [19].

In single-stage reconstruction with a permanent implant, the resected breast tissue is replaced with an implant of appropriate volume. However, it is difficult to obtain symmetry in single-stage reconstructions, and the rate of complications (such as infection, skin necrosis, and implant exposure) is higher than with two-stage methods [15].

The most commonly practiced form of implant breast reconstruction today is two-stage reconstruction [20]. Breast reconstruction can be performed immediately after mastectomy or be delayed for months or years, but immediate operation offers many advantages over delayed operation. First, there is an important psychological benefit for the patient: they can leave the hospital with less physical evidence of the cancer pathology, which helps them maintain their bodily integrity [21]. Furthermore, the aesthetic result tends to be better due to the cooperation of the surgeon who performs the mastectomy. Preoperative surgical incision planning with preservation of the maximum possible amount of healthy skin tissue and muscle increases the possibility of obtaining a natural breast form [2]. However, immediate reconstruction requires a longer period of general anesthesia and increases the risk of infection and hemorrhage, wound size, and healing time [22]. Delayed reconstruction, on the other hand, allows the patient additional time to consider her restorative options.

Reconstruction can be performed after completion of systemic cancer treatment. If so, it will be known if the patient received radiotherapy, and there will be shorter hospitalization and recovery times because the reconstruction does not occur at the same time as the mastectomy. Less dissection is performed, and the risk of skin necrosis is greatly reduced. In this situation, the woman can choose the timing of her surgery and plan for the inevitable interruptions in her daily life with consideration of personal, professional, and family responsibilities. Disadvantages of delayed reconstruction include additional surgery and the negative psychological effects from suffering from body disfigurement for a period of time [20, 23].

The advantages of expander-implant techniques for breast reconstruction also include minimal morbidity compared with the donor site damage with autologous flap reconstruction techniques and the saving of surrounding skin tissue flaps, which remain available for use in different reconstruction techniques [13, 24].

A technical modification to TE-based breast reconstruction is the use of the acellular dermal matrix (ADM) of either human or bovine origin, which allows creation of the submuscular pocket by mobilization of only the pectoralis major muscle [25, 26]. The use of ADM provides numerous advantages over the conventional technique, but there are also potential disadvantages, including higher cost [27]. More recently, autologous dermal grafts have been proposed as an alternative to ADM [28, 29].

The ADM, which may be of fetal bovine, porcine, or human cadaver origin, acts as a “pectoralis extender,” covering the inferolateral portion of the TE and obviating the need for elevation of the serratus anterior muscle, the pectoralis minor muscle, and the rectus abdominis fascia [25, 30–35]. The ADM is typically a 8 × 16 cm sheet of dermal matrix that is sutured to the detached pectoralis major muscle edge and functions as a sling or hammock for the TE. Advocates of ADM point out the many advantages deriving from its use, for example, larger pocket size, higher intraoperative fill volume (even double) [36], increased expansion and enhanced definition of the lower pole, resulting in more natural shape and ptosis, less lower pole rippling, increased control over the IMF and the lateral mammary border, reduced postoperative pain, faster postoperative expansion, and lower capsular contracture formation [37–50]. However, these aesthetic advantages have mostly been accepted on the basis of empirical or anecdotal evidence [51–53]. Some authors have reported increased early complication rates (hematoma, seroma formation, and infection) with the use of ADM [42, 54]. Moreover, in a recent review, Kim et al. [55] reported acceptable complication rates of 8.6%–19.5% after ADM breast reconstruction.

Autologous fat grafting is an increasingly popular technique used in reconstructive surgery of the breast. Autologous fat grafting can be used for simple, aesthetic augmentation of the breast, correction of breast asymmetry, and correction of breast deformities, as an adjunct or primary tool in breast reconstruction, and for soft tissue coverage of breast implants [56]. An increasing number of authors proposed that lipofilling could improve the outcomes of total or partial reconstruction in breast cancer patients [56–58].

Although several teams have used repeated lipofilling sessions for total breast reconstruction, most authors consider that the lipofilling technique is indicated for the local improvement of small defects or asymmetry only [58–60].

Most recently, some authors have proposed the preoperative use of vacuum-based external tissue expander (i.e., Brava device) followed by autologous fat grafting, like an alternative to partial and total breast reconstruction [61–64]. After some weeks of Brava expansion, the breast volume increased by 100 to 300 percent and the authors diffusely grafted the breasts with 100 to 400 ml of lipoaspirate. The addition of Brava expansion before autologous fat grafting leads significantly to larger breast augmentation, with more fat graft placement, higher graft survival rate, and minimal graft necrosis. The device was well tolerated by patients, with satisfying aesthetic results; however, they experienced a higher incidence of skin complication, in particular in irradiated patients [61–64].

## 2. Materials and Methods

### 2.1. Surgical Technique

The first step is preoperative planning, during which breast dimensions, NAC position, and the areola to inframammary fold distance are evaluated to establish the ideal breast size and ptosis degree. During the evaluation, the possible reconstruction options are discussed, not only for the affected breast, but also for the contralateral breast. Augmentation, reduction, and nonintervention are all therapeutic possibilities.

The complexity of the decision-making process that precedes breast reconstruction surgery has increased due to the great range of reconstruction strategies available today [65–67]. Less-experienced surgeons can use algorithms, flow charts, and nomograms to help them plan surgery [68–70]. More recently, surgical planning systems have been devised as well as virtual simulator systems to train surgeons outside of the “apprenticeship model” [71–73].

The size of the device is based on breast width and size and the contralateral breast shape, but one must also consider patient wishes for the contralateral technique [13]. The surgeon must be aware that the patient's main concern is her attractiveness after breast reconstruction and that the final breast size should correspond with body size and influences patient satisfaction with her self-image [74].

The technique requires two stages: a temporary device (tissue expander) is placed in a submuscular pocket during the first operation if immediate breast reconstruction is performed and at the first stage if delayed reconstruction is performed. After sufficient healing has occurred, the expander is filled with saline in a serial fashion over several weeks or months to the desired volume. Then, after there has been sufficient expansion, the patient returns to the operating room for removal of the expander and replacement with a permanent saline or silicone implant [2, 8].

#### 2.1.1. First Stage

When the mastectomy is finished, it is possible to start with the reconstruction step. If the mastectomy flap is deemed viable, the surgeon can proceed with immediate reconstruction by creating a pocket for a tissue expander. Either a partial or a complete submuscular pocket is created, with the same dimensions as the selected expander. The chosen expander should have the same base width and height as the contralateral breast. It is important to avoid implant visibility or exposure; thus, complete soft tissue coverage of the expander must be ensured.

After mammary gland removal, a new evaluation of the quality of the remaining skin and muscle is performed. It is important to redefine the edge of the pocket and the inframammary fold position, comparing them with the size of the expander previously chosen.

The patient is positioned on the operating table with the upper arm adducted to 60° to obtain complete relaxation of the pectoralis major and to facilitate muscle dissection. The contralateral breast should be visible in order to obtain the best possible symmetry during reconstruction. Pectoralis major dissection starts from its lateral edge, followed by the superior, medial, and inferior borders. Inferolaterally, the aponeurosis of the anterior rectus muscle and the external oblique is exposed. Finally, the muscle is dissected from its sternal attachment at the level of the second intercostal space. At this point, the pectoralis major can be elevated to create a superior pocket. Dissection of the pectoralis major muscle is easy and minimally traumatic to the patient.

**Figure 1:**
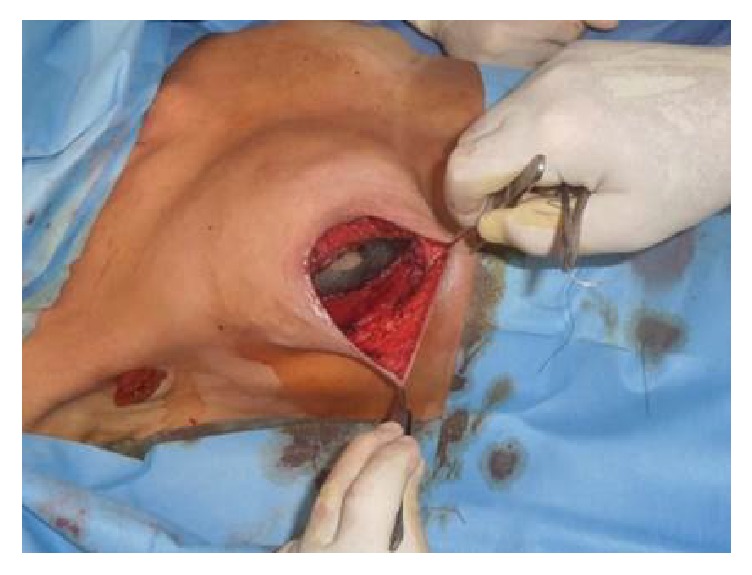
Implant placement into the submuscular pocket.

The traditional approach to creating a pocket for the expander and implant is to optimize muscle coverage (Figure 1) [75]. Accordingly, both the pectoralis major and the entirety of the serratus are initially raised [76]. However, raising the serratus muscle completely off the ribs leaves a surface overlying the chest wall that is painful and not ideally suited for the sutures that would subsequently define the inferolateral aspect of the reconstructed breast. An alternative is the creation of a musculofascial pocket in which the whole pectoralis muscle composes the superior portion but laterally includes only a part of the serratus muscle with its entire overlying fascia. This dissection allows adequate space for the tissue expander without the risk of it folding on itself [77]. A drain should be placed into the pocket before the expander. It should be immersed in iodopovidone or antibiotic solution, correctly oriented, and placed into the mastectomy cavity. Finally, the pocket is closed with reabsorbable interrupted sutures [13].

The expander is completely evacuated of any retained air and then inflated with saline up to 20%–30% of its final volume to facilitate its insertion into the pocket. After the intervention, the expander is inflated to approximately 50% of the total volume to expedite the expansion process and decrease the risk of postoperative hematoma and seroma. The increase in intraoperative fill does not increase the rate of mastectomy flap failure [78, 79].

Expansion time may vary between 3 and 7 months. Generally, the tissue expanders used for breast reconstruction are anatomically shaped, and the lower pole can be further expanded. Full-projection of the tissue expanders helps recruit upper pole tissue to highly expand the lower pole, giving a naturally ptotic appearance to the reconstructed breast [80]. Overexpansion by approximately 30% is needed to obtain an acceptable degree of breast ptosis [8, 15].

#### 2.1.2. Second Stage

The second stage is commonly performed at least 6 months after the end of tissue expansion [81]. The mastectomy scar is normally removed and new access is created at the same site. Subcutaneous and muscular dissection is performed to enter the pocket and remove the tissue expander, previously emptied.

An important part of the exchange procedure is setting the inframammary fold and the inferolateral extent of the implant pocket. The inframammary fold is the most critical visual landmark of the breast, and the entire final reconstruction is based on this landmark. After the expander is removed, the inframammary is set using a suture that fixes the deep dermis to the anterior chest wall. Needles are inserted into the pouch through the skin to mark it. The shape is reconstructed with several sutures, creating an aesthetically pleasing curve inferolaterally.

In addition to defining the inframammary fold, the sutures create a fixed edge that prevents the implant from migrating laterally. Radial capsulotomy is more commonly performed to allow centripetal release of the breast. However, circumferential capsulotomy allows obtainment of an increase in projection from 1 to 7 cm [36, 77].

The permanent implant can be filled with saline solution or silicone gel; the US Food and Drug Administration approved silicone in 2012. The surgeon can choose either silicone-filled shaped implants or round implants to promote a natural aesthetic outcome [82]. The most common implant type currently used is a prosthesis filled with a very dense silicone gel, called highly cohesive. The firmness of the filler material prevents capsular contracture and preserves the original shape of the implant.

When the selected prosthesis is inserted, the appearance and symmetry with the contralateral breast are checked with the patient in the sitting position [13]. Finally, the wound is closed with double-layered absorbable suture material.

## 3. Results

Mastectomy is a destructive but essential intervention for women with breast cancer.

Tissue expansion breast reconstruction is a safe, reliable, and efficacious procedure with considerable psychological benefits since it provides a healthy body image [78, 79, 83].

**Figure 2:**
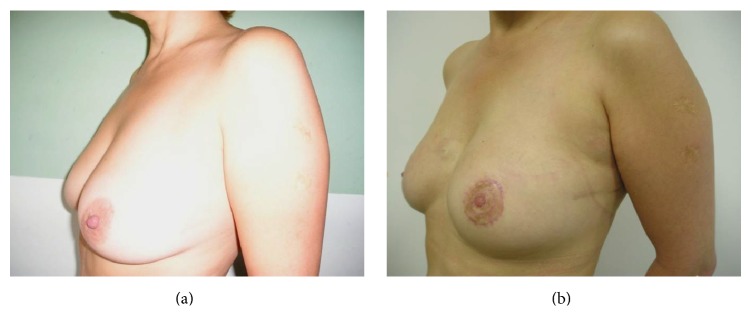
(a) A 45-year-old patient before mastectomy and reconstruction surgery. (b) The same patient after tissue expansion and implant reconstruction, showing the final result.

More realistic reconstruction, even in patients with aggressive surgical resections, can now be obtained due to advances in cohesive-silicone implants. Even though autologous breast reconstruction provides a better cosmetic outcome with a more natural-appearing breast, tissue expander-based reconstructions have the advantages of shorter operative times, faster recovery, and lack of donor site morbidity. Moreover, the aesthetic result obtained with tissue-expander reconstruction is considered more than acceptable by most patients (Figure 2) [84].

Acquired contour deformities after breast reconstruction are relatively common and occur independent of the technique used, presenting a frequent therapeutic challenge to reconstructive surgeons [85]. The transplantation of autologous adipose tissue is a simple and effective solution for many immediate or delayed postoperative complications, such as for defining the inframammary fold, correcting profile asymmetry, or filling neobreast defects. In addition to adipocytes, cell types such as fibroblasts, smooth muscle cells, endothelial cells, and preadipocytes compose the fat graft. Stem cells isolated from lipoaspirates have demonstrated in vitro adipogenic, chondrogenic, osteogenic, and myogenic lineage commitments [86, 87] and have shown differentiation into pancreatic cells, hepatocytes, and neurogenic cells [88–90].

The stromal vascular fraction of the fat graft has an important regenerative function and is also responsible for the paracrine secretion of various factors such as VEGF, HGF, and TGF-*β*. These factors are released in response to different stimuli, including hypoxia. They strongly influence stem cell differentiation, induce angiogenesis and tissue remodelling, and stimulate wound healing [91, 92].

There is no standardized protocol for isolating Adipose-Derived Stem Cells (ASCs) for clinical application. In fact, in the literature, it is possible to find different methods described based on fat centrifugation with or without the addition of collagenase or other enzymatic reagents [93–105].

## 4. Conclusion

Tissue-expander reconstruction is a safe and effective technique chosen by many patients. It is an attractive option due to the lack of a donor defect, reduced recovery time and potential morbidity, and the fact that it allows women to exercise choice in the size of the reconstructed breast.

## Additional Points

This paper is a description of the important psychological effect of breast cancer surgery, a focus on the most common surgical reconstruction technique and analysis of esthetic and psychologic results.
